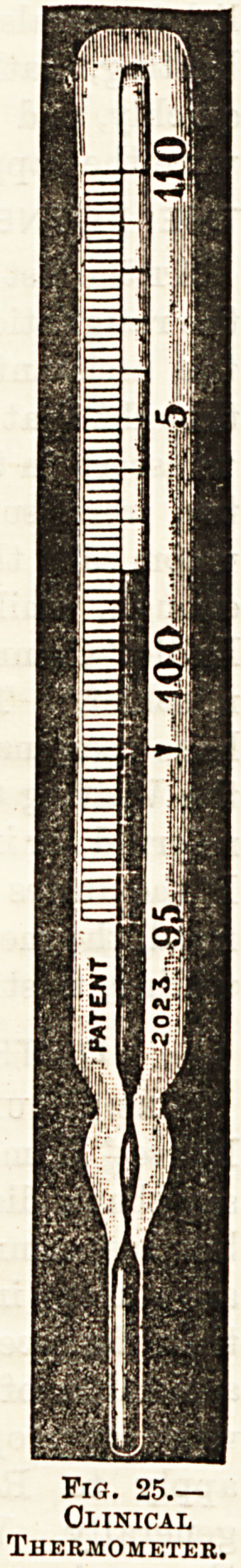# "The Hospital" Nursing Mirror

**Published:** 1900-09-15

**Authors:** 


### "The Hospital" Nursing Mirror.

Being the Nursing Section of "The Hospital."

[Contributions for this Section of "The Hospital" should be addressed to the Editor, The Hospital, 28 & 29, Southampton Street, Strand, London, W.C., and should have the word "Nursing" plainly written in left-hand top corner of the envelope.]

### Notes on News from the Nursing World.

### OUR CHRISTMAS DISTRIBUTION.

As usual at this time of the year, we invite the kind friends who put it into our power to make a distribution of useful gifts at Christmas to the patients in hospitals and infirmaries to start upon their good work as soon as possible. We know by experience that the appeal will be cheerfully and liberally responded to. There is no need to indicate the character of the articles most appreciated. The nurses find that warm and serviceable garments of every kind are always in request, while the more numerous they are the greater will be the pleasure afforded.

### THE WANT OF MILK IN SOUTH AFRICA.

One of the nurses who has lately returned to South Africa in the "Simla," writing to us, touches on a matter which has, perhaps, been somewhat lost sight of. She says that South Africa has suffered greatly during the past two or three years from rinderpest, which has carried off the greater part of the farmers' stock, so that milk, which is so necessary in enteric and dysentery, has not been plentiful nor easy to get, as at ordinary times. She points out that it is difficult to know what to use as a substitute. With regard to complaints of mismanagement, she says: "From what I hear I think everything possible has been done for the sick. Of course, there are always some who will grumble, no matter how well they are treated, but the majority are satisfied. There is no doubt that want of railways has hindered much that might otherwise have been done. As to the hospital ships, the 'Simla,' on which I went home, was supplied with everything that could be needed, and at each port we touched, such as Cape Town, St. Vincent, and Southampton, the 'Absent-minded Beggar' was waiting with warm clothing and little luxuries in the way of pipes, tobacco, &c."

### A VOICE FROM BIRMINGHAM.

Mr. Leedham-Green, of Birmingham, who relieved Mr. Treves at Chieveley Hospital when the latter was attacked by dysentery, declares that the nursing in the base hospital at Maritzburg, where he arrived when the wounded were being brought in from Spion Kop, was "quite inadequate." More nurses and more orderlies, he says, were needed than could be obtained. In illustration of this, Mr. Leedham-Green mentions that in one block he had 100 beds, the occupants of which were suffering for the most part from bullet wounds, many of them being reduced to absolute helplessness. "During the day one sister and two orderlies were in attendance. During the night not a single attendant was left with the patients. If they needed anything they had to crawl about and help themselves; those who were unable to do so had to appeal for the help of their comrades around them." "The nurses," adds Mr. Leedham-Green, "worked admirably throughout the Natal campaign, taking very little rest and working in many cases almost day and night. The orderlies, too, were very attentive, and were constantly working overtime without a grumble." At Chieveley, on the other hand, he says, the arrangements were admirable. "The patients were well looked after, and the nursing was excellent."

### A MISTAKE IN THE SEX.

The *Western Mail*, quoting a letter from a member of the Army Nursing Service Reserve in our issue of September 1st, describing the writer's experience in the convalescent officers' quarters in Cape Town, makes an amusing mistake. Our contemporary in its comments assumes that the members of the service are men, a hypothesis which in the face of the quotations given must have been rather bewildering to the readers of the *Western Mail*. They must also have been surprised to learn that the Americans "for some occult reason called the quarters a sanatorium." Our correspondent wrote, "This sanatorium is managed by Americans, who for some occult reason call the place a 'sanitarium.'"

### THE LIFE OF A HOSPITAL NURSE.

While a discussion is going on in the "Nursing Mirror" respecting the, apparently, always interesting question of how far nurses should engage in menial work, our attention has been directed to an article in the *Humanitarian* on what is described as "An Unknown Side in the Life of a Hospital Nurse." The author, Miss Elizabeth French, clearly intends to convey the impression that hospital nurses, as a class, are overworked and badly treated; for before she narrates the experiences of "Nurse F.," she says: "The system of training varies little in different hospitals, so that in giving a nurse's experience of one it may be taken as a general type of them all." It is because of this statement that we think it necessary to allude to the article. There is always a possibility of an individual nurse in a particular hospital having solid cause for complaint, since neither hospital systems, nor hospital matrons, any more than hospital nurses, are perfect. But, even assuming that the facts are as set forth in Miss French's article, and that at two hospitals, somewhere in the United Kingdom or elsewhere, the strain of work imposed is too heavy for an ordinary woman to bear, it is most unfair to attempt to tar other institutions with the same brush. The life of a hospital nurse as usually known, and not as portrayed by a writer, who furnishes no clue by means of which the accuracy of her details can be put to the test, is not a bed of roses. But the picture of a strong, healthy probationer having her health "shattered" in one hospital in a few months, and receiving her death-blow in another in "about a fortnight after she had begun her work," is a travesty of hospital life. How little the author is really acquainted with its conditions is indicated by her remark that "if, in the prevailing system of working our hospitals, one-hundredth part of the care and consideration that is lavished on the patients were given to those who must be able to attend to them then the nurses' lives would not be so unscrupulously, sacrificed." This is wholesale slander, and, though some excuse may be made for the distorted vision of a writer who has lost a friend, or a relative, in the pursuit of a profession she hoped to adorn, it is sufficient to render her an untrustworthy witness.

### ST. THOMAS'S NURSING HOME.

The following appeared last week in the *Western Mercury*, a paper of large circulation in Plymouth and the West of England:—

"I am sorry to hear that the Charity Commissioners are showing a disposition to break down one of the most useful institutions in London—I refer to the St. Thomas's Nursing Home, attached to the hospital. These paying wards are under the direction of the same medical staff as the free or main portion of the hospital. The technical ground of objection on the part of the Commissioners is that, as the institution is established by charitable endowment, no charge should be made in any wing. There are, however, many people tenderly reared and with small means who find the greater privacy and little comforts of the place a great convenience, and they would be quite unable to bear the cost of nursing in the West-end private homes in which doctors are interested. In St. Thomas's one finds poorer middle-class people from all parts of England, and they speak very highly of the treatment."

Upon inquiry at St. Thomas's Hospital the authorities, as we anticipated, inform us that nothing is known of the matter there. Moreover, it may be pointed out that the Charity Commissioners were fully cognisant of the arrangements for the nursing home when it was originally started. The only possible foundation for the statement must have been that the home has lately been closed for a month for the usual cleaning, painting, and redecorating. It was, however, reopened a week ago, and is not in the least likely to be closed again, except for a similar purpose.

### THE ALLEGED "STRIKE OF NURSES."

With reference to the alleged strike of nurses at St. George's Infirmary, Fulham Road, which has been reported in a London daily paper, we are able to state that, as a matter of fact, nothing of the kind has occurred. It is quite true that there have been several resignations since last June. One nurse obtained promotion in another infirmary, two resigned because of ill-health, one left on account of the death of her mother, and five resigned in order to get married. It will be admitted that the reasons for these resignations, especially the last, are perfectly valid. In consequence of this, and for other causes, the staff has been increased by an unprecedentedly large number of candidates for probationerships, and as the natural process of selection s proceeding during the two months' probation, there is more in-coming and out-going than usual amongst the staff. This explanation is necessary, because the nurses at St. George's Infirmary feel that the value of their certificate is being depreciated when it is represented that they are the victims of an ill-managed institution. They are, we are told, satisfied that when the new home is ready for occupation—which it will be in the near future—such grievances as they now have will be done away with, and no idea of resorting to a proceeding so entirely indefensible as a strike has entered their minds.

### A PENSION FUND FOR AUSTRALIAN NURSES.

A brilliantly successful conversazione was given on Friday evening, July 20th, in the Town Hall, Sydney, by the Mayor and Mayoress, Sir Matthew and Lady Harris, to meet the members of the Australian Trained Nurses' Association. About 2,000 invitations were issued, and quite 1,500 were accepted. The nurses, in their house uniforms, gave an appropriate tone to the highly representative assemblage. The hall was superbly decorated with draped flags and a profusion of palms and other foliage. This first annual gathering of the trained nurses was intended not only as a social entertainment, but as a means of inaugurating a Pension and Benevolent Fund in connexion with the association; and during the evening the President, Dr. Norton Manning, having explained its aims and objects, Sir William Lyne, Premier of New South Wales, advocated the necessity for starting a pension fund. His speech, and that of Mr. John See, urging the public to liberally respond to the request for subscriptions, were received with applause, and we are pleased to learn from a Sydney correspondent that the actual formation of a pension fund is practically beyond doubt. One gentleman has given £21 towards it, and though it is hard to get money just now owing to the people in New South Wales having had so many calls upon their generosity lately, the movement commands general sympathy. The great success of the conversazione must also tend to make the wants and claims of the Trained Nurses' Association more widely known amongst the residents of Sydney who employ nurses, so that in future the qualifications of the nurses engaged will be more carefully investigated.

### NURSING IN A WELSH INFIRMARY.

A nurse in a union infirmary in Wales confirms from her own experience the urgent necessity for reform in workhouse nursing. We give the substance of a letter we have received from her:—

"Three weeks ago I commenced duty as superintendent-nurse at a workhouse infirmary of sixty beds, forty-three of which were and still are occupied. The staff consisted of a temporary midwife and an assistant nurse, trained here. The latter had resigned before my appointment, and left a week after my arrival. Her place was filled by an assistant nurse with a little training. A week after she arrived the temporary midwife, who was on night duty, left. The newly-appointed assistant nurse had, of course, to go on night duty, and I was left alone on day duty. The doctor said it was unnecessary to get an emergency nurse, as the guardians were advertising in the local papers for another nurse. The first morning I was left alone there was a confinement, two of the wardsmen had a fight, and another had a fit. For three days I was quite alone, the doctor not putting in an appearance, but on the fourth day the matron very kindly sent a house-nurse to help me in the morning, and enable me to go out in the afternoon. Sunday I was alone all day with two women in labour, and about seven p.m. one was safely delivered, and the other half an-hour later. From Wednesday till now, Monday evening, the doctor has been in once. The master and matron are both very anxious that I should be comfortable, but against such odds how can I?"

Comment is superfluous, except that it is pleasant to hear of the master and matron of a workhouse doing their best to make things pleasant for the nurse.

### A NATIVE HOSPITAL IN MAURITIUS.

A nurse working in Mauritius sends us the following account of her holiday: "I obtained permission to leave the camp for a few days, and in company with a friend set out for a small village on the coast called Mahebourg. We took up our residence in a bungalow close to the sea, which had been lent to us, and the caretaker, an Indian, cooked our food and did o[illegible]

marketing. Mahebourg is a very pretty place, with a long range of mountains on one side. It was at one time the most important place in the island. The entrance to the bay is protected by five small islands, on one of which there is a lighthouse. The hospital for native patients contains 102 beds, divided between men and women. There are six huts—one large one for women, three small ones for men, one for maternity cases when required, and also one for isolating infectious cases. In one corner of the compound a separate hospital had been erected for cases of 'plague,' and in another corner a 'segregation camp.' Fortunately, it had not been needed, but it was there in readiness. The patients appeared comfortable, but 'trained nurses' were conspicuous by their absence, and everything was very different to what one is accustomed to see in English hospitals. We spent the days very pleasantly in boating, bathing, and fishing; they passed all too quickly, and when the time came for us to return, we were the happier and better for the change."

### THE TOTNES GUARDIANS AND THEIR NURSES.

At the last meeting of the Totnes Board of Guardians the resignation of the superintendent nurse and also of the assistant nurse were announced. The Chairman thought that the Visiting Committee should endeavour to ascertain the reason for the continued changes; and two were suggested by subsequent speakers, one of whom said that the diet of the nurses was not varied enough, while another ascribed the friction to the Ladies' Committee visiting the house, and "stirring up mischief." This is a serious allegation, and should not have been made if it cannot be fully sustained. Perhaps the Visiting Committee, to whom the matter has been referred for inquiries, will be able to get at the truth. If the duties of the Ladies' Committee are clearly defined, the members should not be able, even if they are willing, to stir up friction.

### DISTRICT NURSING AT RICHMOND.

Mrs. Boultbie, the Superintendent of the Victoria Nurses' Home at Richmond, Yorkshire, writes to us to say that a district nurse has not only been appointed, but has commenced her work, and that "fresh cases are coming in daily." She also wishes to explain that the reference, at a recent meeting in the Town Hall, of a member of the Corporation, to people "dying from want of proper nourishment and treatment," did not apply to Richmond especially, but to the world generally. We are glad to learn that, while it is clear the services of a district nurse were much needed in the town, Richmond had not obtained the unenviable reputation which the remarks of Mr. Hodgson, as reported in one of the local papers, suggested.

### A POINT OF PROCEDURE.

It is very important that when committee meetings of nursing associations are held all the members of the committee should receive proper notice. At the annual meeting of the Aberlour Nursing Association, Provost Jupp said he wished to resign, as questions had been dealt with unknown to himself and some other members of the committee. It then transpired that the ladies of the committee had been in the habit of holding meetings without formal notice, the gentlemen going to them if they happened to hear of them and cared to attend. This slip-shod method it was very properly decided to remedy, and it being understood that in future all members of the committee should be apprised of every meeting convened to deal with other than routine things, Provost Jupp consented to remain a member. It is satisfactory to learn that the reserve fund of the association stands at a little over £50, and that the nursing is greatly appreciated.

### TUNSTALL TOWN NURSES' FUND.

Satisfactory testimony was given the other day at at a meeting of the Tunstall and District Hospital Saturday Committee, as to the nature of the work done by the Town Nurses' Fund, and we do not doubt that its treasurer was justified in assuring the meeting that any money voted to it would be very well spent. But the managers should take care in future that there is no dilatoriness in issuing the balance-sheet. A complaint was made by a member of the committee that it was five years since he had seen a balance-sheet, and though the treasurer replied that the last was issued in March, 1899, he failed to justify the non-appearance of one in March, 1900. His plea was that "subscriptions were behindhand." But balance-sheets should not be behindhand whatever may be the case concerning subscriptions. We can quite understand that the fund itself has suffered from procrastination in preparing a financial statement. The need for further support should rather have been a cause for punctuality than an excuse for delay.

### THE GWESPYR VILLAGERS' NURSING CLUB.

Allusion was made in a recent issue to the Gwespyr Nursing Club, originated by Lady Mostyn. We now learn that the services of Nurse Parsons, one of the Queen's Jubilee nurses, have been secured, and a residence provided for her in one of the lodges on the Talacre estate. Sir P. Mostyn has given £60 as the nucleus of a club fund, and he and Lady Mostyn contribute £30 yearly to the salary of the nurse. The Queen's Jubilee Institute also grants £10 for the first year.

### THE S.S. "TEMPLEMORE."

One of the nursing sisters on board the "Templemore," which arrived the other day from South Africa, writes to us to say that she cannot speak too highly of the voyage home, that everything was very comfortable and clean, and that the captain did everything for the comfort of both patients and nurses.

### SHORT ITEMS.

The eighth annual report of the Wilton Beacon Nursing Association, of which Lady Margaret Bickersteth is honorary secretary, shows that the nurses attended 128 cases, that the number of benefit subscribers has increased, and that a debt of £13 14s. 10d. has been cleared off, and replaced by a balance in hand of £33 1s. 7d.—The principal item of interest to nurses in the programme at the Conference of Women Workers, to be held at Brighton next month, is "The Work of Women on Hospital and Other Boards," which is down for discussion on the afternoon of Wednesday, October 24. A paper will be read by Miss Louisa Stevenson, of Edinburgh.—The marriage of Miss Jennie Speatley, eldest daughter of Mr. John Speatley, of Luton, and a nurse for nine years on the staff of the Trained Nurses' Home, Wakefield, to Mr. Colin C. Roberts, of Wakefield, took place at Luton on Thursday last week. Among the numerous handsome presents to the bride was a silver-mounted purse, containing gold of the present currency, from her former colleagues at the Wakefield Home.

### Lectures on Nursing for Probationers.

By E. MacDowel Cosgrave, M.D., &c., Lecturer to the Dublin Metropolitan Technical School for Nurses.

### XV.—TEMPERATURE.

The temperature of the body in health is 98·4 deg. F. (37 deg. C.); it is always the same whether the surrounding air is hot or cold, whether we feel warm or chilled. The heat of the body is caused by the oxidation or burning of the tissues and of the materials taken up by the blood from the digestive organs; heat is formed by the oxidation of the body just as surely as it is formed by the oxidation of coal in the fire.

The muscles are the most important source of heat, but large active organs, such as the liver and brain, contribute a good deal, and all the body helps, as every living tissue is always producing some heat.

The oxidation that takes place in the body has two uses; about one-sixth is devoted to doing work, the remaining five-sixths appearing in the form of heat. The heat is distributed throughout the body by the blood, and the regulation of the temperature is carried out to a great extent by altering this distribution. When heat has to be economised the blood collects in the deeper parts; when it has to be given off the blood flows to the skin. The body is constantly giving off heat; most escapes by the skin, but the breath also gives out a good deal. The regulation of the heat of the body depends upon a due balance being preserved between these two processes—heat production and heat loss. When the amount of oxidation is increased more heat is given off from the body, and when oxidation lessens the amount of heat given off diminishes; in each case the temperature of the body remains the same. So, when exercise is taken, though the muscles are called into activity, and oxidation is greatly increased, the temperature of the body is not increased, as the blood vessels of the skin dilate, the hot blood comes to the surface, and the extra heat escapes. As long as the air is colder than the blood this relaxation of the vessels and the consequent flow of hot blood to the skin will cool the body, as some of the heat passes out into the air; this radiation, however, occurs more in cold weather than in hot, and when the air is as hot as the blood the giving off of heat in this way practically stops.

It is the giving off of perspiration that assists radiation, and allows the temperature to be kept regular no matter how hot the surroundings. When water changes into vapour a great deal of heat is absorbed and stored up in the vapour. This can be tested by pouring a little ether or spirit on the hand and waving it about, when well-marked coldness will be felt. Indeed the skin is sometimes frozen by the rapid evaporation of ether or of chloride of ethyl, so that operations can be painlessly performed. So powerful is the cooling property of perspiration that it is possible to remain for some time in a room, or oven, where the air is very much hotter than the human body. This is done, for example, in the ordinary Turkish bath. Cold causes slightly increased oxidation, so more heat is formed in cold than in hot weather. There is also, as we have seen, economy of heat, as cold causes the blood vessels to contract and drive the blood into the deeper organs, where its heat is preserved, and, as a result, perspiration almost ceases.

Our clothes help to retain heat and prevent its loss by radiation; wool, being a bad conductor, retains heat much better than cotton or linen. Wool has another advantage, it absorbs vapour without allowing it to condense into moisture as cotton and linen do, and so wool does not allow the chilly feeling after exercise that is felt if cotton or linen is worn next the skin.

In illness the temperature often varies from the normal standard, and it is raised in fevers, inflammations, &c. It falls in early convalescence after an acute illness, and in collapse. The range of temperature usually met with in illness is from 95 deg. to 105 deg. Anything above or below this is dangerous; indeed, if the temperature remain for any length of time at 105 deg. it is dangerous, and in fever whenever the temperature rises above 103 deg., sponging, or some such other means of reducing the temperature is resorted to. Any temperature over 100 deg. is called pyrexia or fever, whilst a temperature from 105 deg. to 110 deg. is called hyperpyrexia. The latter is very dangerous, as a temperature of 107 deg., if not speedily reduced, will rapidly cause death.

Fig. 25.—Clinical Thermometer.

The reason the temperature rises in fevers and in inflammation is that there is extra burning, and consequently more heat production, and dryness of the skin and mucous membrane, and so diminished giving off of heat. At the commencement of a fever the formation of heat is greater than the giving off, and so the temperature gradually rises. After a time they get equal, and the temperature remains high; gradually the giving off becomes greater than the formation, and then the temperature falls once more to normal. The average height at which the temperature remains is important, as a continuously high temperature is more dangerous than one which is high at one part of the day and low at another. A patient has no idea of his own temperature; the feeling of heat depends entirely upon the nerves in the skin. When a rigor occurs the skin is pinched and blue, and the patient is shivering and complaining of cold, yet his temperature may be 104 deg. or 105 deg.

In the first stage of ague the temperature is high, and yet the patient feels cold; in the second stage the blood rushes to the skin, the temperature rapidly falls, and yet the patient complains of heat, tosses aside the bedclothes, and asks to have the window opened. The hand of the observer also is unreliable, as its own condition affects its judgment. Thus to a cold hand tepid water will feel warm, while to a warm hand it will feel cold. So a clinical thermometer is used to take a patient's temperature. This has two peculiarities—one is an index which records the temperature; the other is that it has on its scale only the fifteen degrees, from 95 deg. to 110 deg., through which the human temperature varies, so extra space can be given to these degrees, and even so small a difference as a tenth of a degree can be read. Before using the thermometer the index must be shaken down below the normal point, which is marked by an arrow head. The temperature is generally taken in the axilla, in restless children it may be taken in the groin. If the skin is pinched and blue, as in a rigor or in collapse, the internal temperature should be taken; this may be done in the mouth or in the rectum. The internal temperature is generally about a degree higher than the skin temperature. The bulb of the thermometer must be well surrounded by flesh, so when the bulb has been placed in the axilla the arm should be drawn to the side, the elbow being slightly raised towards the front of the chest. If there is perspiration the axilla must be carefully wiped out. The thermometer should be left in for five minutes.

### Six Months' Nursing at the Princess Christian Hospital.

By a Member of the Army Nursing Service Reserve.

A correspondent, writing from Pine Town Bridge, Natal, under date of August 13th, says: —

It is more than five months since the Princess Christian Hospital was despatched from England, but this is the first time we have had leisure for writing more than the weekly letters home. The hospital was intended for 100 patients, and six sisters of the A.N.S.R. were sent out, namely, E. C. Laurence (of Guy's Hospital), superintendent, E. Atkins, M. Leng, F. Baker, D. Snell, and E. Fisher. Our destination was uncertain when we sailed on March 3rd, and at Cape Town we found that quarters had been taken for us in a boarding-house, so we all bundled up the town with our baggage, &c., to hear an hour or so later that we were to rejoin our ship, the "Tantallon Castle," and proceed to Durban. We had a very rough trip up the coast, and when we reached Durban the place was so full we had to wait on board two days before any quarters could be found for us. We were then dropped over the ship's side in a basket, in the approved fashion of landing at that port.

### The Site.

We then learnt that we were to pitch our tents (or rather to build our hospital) at Pine Town Bridge, a very fine site having been most kindly lent to the Government by Mr. Stevens, of Durban. We had hoped to go further up country, and Mooi River had been suggested; but the general opinion seemed to be that, with the winter coming on, it would be too cold for sick troops up there, so we were lucky to get this site in beautiful fruit-growing country and only an hour by rail from Durban. As we found we should have to stay at least a week in Durban we employed our time by being inoculated against enteric, not having been able to get done on board ship. It is not a pleasing experience—a few days of high temperature and intense pain; in fact, typhoid in a very concentrated form.

### Inoculation and Enteric.

But later on we were thankful we had all been done, as our hospital was soon full, nearly all being enteric cases, and I think I should not be far wrong in saying that this is about the only hospital in South Africa where none of the sisters nor medical officers has contracted the disease. I regret that I cannot say the same of our orderlies; seven of them having had the disease, and one died of it.

There were three brick buildings already up on the site, but when we arrived they were full of refugees. As soon as other quarters could be found for them we came up and helped with the unpacking and checking of furniture, crockery, linen, &c. Mr. Mosely had brought out four large pavilions to hold twenty-five beds each, but it was decided that we were to be enlarged to a two hundred bed hospital, so the Government sent up material to build five more small wards for sixteen beds each, one of the buildings already up being turned into a small officers' ward of ten beds. All the buildings are iron and wood; the four pavilions being lined with a pretty green canvas, and having a cosy little sister's sitting-room (besides a pantry, linen-room, and bath-room). These are considered superior to the Government wards, though the latter are just as comfortable from the patient's point of view.

We opened unexpectedly (before any of the iron buildings were ready) on Easter Monday, April 16th, when a troop train was thrown off the line about two miles from here. Two men were killed and sixteen mules, and we admitted three men of the A.S.C. and two Kaffir mule drivers, all rather badly hurt, though all recovered.

### The First Patients.

Our first batch of sick arrived early on the morning of April 28th, fourteen officers and sixteen men, nearly all enteric cases, and nearly all very ill. Hardly any of them had slept on a bed since they went up to the front in November, and their delight at the comfort of wire mattresses and clean sheets was very good to see. They came down in the Princess Christian Hospital train, which is most comfortably fitted up and arranged, but even so the fatigue to a bad enteric of a journey of more than 150 miles over a rickety, single line railway was very great, and the doctors hardly thought that three or four of them would live through the first night; but with a plentiful use of stimulants they all did, and only one died out of the whole thirty. It was worth while coming out to South Africa just to pull that batch of enterics round, and it was only by sheer hard nursing that we did it. One young officer who had been ill three weeks told me that he had been moved from one camp to another no less than five times, the order to "move camp" always arriving in the evening, when his temperature was highest. When he arrived here his temperature was over 104, and we thought for some days that he would slip through our fingers from sheer exhaustion, but he soon began to pick up, and went home very well.

### Inexperienced Orderlies.

Our orderlies were all men of the St. John Ambulance Brigade, very well trained in stretcher carrying, "first aid," &c., but they had had no previous experience of nursing at all. They were engine drivers, carpenters, tailors, painters, &c.; we had not a single man of the R.A.M.C. amongst them. I cannot say enough for their cheerful willingness over disagreeable tasks, and their keenness to learn and be useful, and now—after more than five months' work—they are becoming really useful and trustworthy nurses. But at first, when the cases were very severe, the sisters practically had to do all the nursing, and they had plenty of hard work acting as their own staff nurse and probationer all in one, the orderlies being of about the same value as a ward-maid in an English hospital.

### Few Wounded.

We have had very few wounded here yet—chiefly enteric and dysentery, with a sprinkling of rheumatism, pneumonia, operation cases, &c. The wards are very comfortably fitted up, and there has been no real scarcity of medical comforts, though the great rush of bad enterics at first taxed our resources considerably. For some time after the 200 beds were in working order there were no more sisters available, so it was pretty "hard going" for our original six.

### Increase of Nursing Staff.

Our staff has since been increased to nine, Sisters Fraser, Bowhill, Smith, and Walker (of the A.N.S.R.) having joined us, and one of our six having gone home as senior sister on one of the hospital ships. Just now there are more sisters arriving in Durban than they know what to do with, so we have four lodging with us for "temporary duty" until they are needed elsewhere. With only about eighty men in hospital, and these nearly all convalescent, we are having a very slack time. We expect some more down very shortly, as they say that all Lord Roberts' sick and wounded will come down this way now that the railway is opened up to Pretoria. Mr. Mosely's time being up for maintaining this hospital on July 20th, he handed it over "all standing" to the Government, so that we are now a military hospital proper. Some of our orderlies left when Mr. Mosely did, and they were replaced by men of the Imperial Bearer Co., chiefly refugees from the Transvaal. Several of them are old and grey, and one feels quite shy about telling them to hurry up over their work when they look at you through their spectacles. Most people seem to think the war will be over in a few weeks now, but there will be work for many of us here for some months yet. The winter is just about over, and summer always brings enteric again up country. Our patients generally come to us in train loads; sometimes thirty stretcher cases are carried in during a couple of hours, at others seventy milder cases. It is always pretty hard work for the first few days after a new batch arrives; many of them have not been able to get any water for washing purposes since they "went sick," and it takes a little while to get the grime off.

### The Hospital Commission.

I expect people at home are anxiously watching for news of the Hospital Commission, and we are very much interested too. It is difficult to say anything shortly on the subject, but I wonder whether people in England at all realise what it means to feed an army like that of Lord Roberts's, when everything has to be brought over 900 miles on a single line railway, nearly every bridge having been destroyed on the last 150 miles, so that the traffic has to be worked over temporary bridges, frequently only a few trucks being able to cross at one time Also this is a most unusually dry season, and it is not uncommon for an engine to have to leave its train in a siding while it struggles on to the next station for water and returns again. An officer of the A.S.C. told me it was almost impossible at one time to get over the railway a single pound more than the men's necessary daily rations. The Boers having planted no crops, there was practically no food supply except that brought up by rail. Then comes a sudden and unexpected outbreak of enteric, the men falling out by the hundred. Is it surprising that tents and all medical comforts were difficult to obtain at once?

### Incessant Trains.

I know on this side, where there is also only a single line from Durban to Ladysmith, and now to Pretoria (and everywhere up country) the line passes within a few hundred yards of our hospital, and it hardly seems as though there is a quarter of an hour, day or night, without a train running past. Several trains every day with nothing but great tanks of water, trains with mules, trains with forage, trains with provisions, trains with building materials, &c., &c. The only marvel is that there have been so few railway accidents. Everyone seems to agree that Tommy has never had so much done for him in any war as in this. At the same time, Tommy has suffered at the front when knocked over by sickness, but I think if people try to appreciate the vast difficulties of lengthy transport, the huge numbers who had to be fed, and the suddenness of a bad outbreak of enteric, to cope with which a large staff of skilled nurses with nursing appliances were essential and were unobtainable at first, they will be less inclined to blame the hard-working R.A.M.C., who have done such grand service all through the war. If hospitals at the base were badly managed and the patients were uncomfortable someone must be to blame, and they wi[illegible] surely get blamed, for I speak from experience when I say that both at Durban and Cape Town everything needfu[illegible] the comfort and well-being of their patients could easily be obtained if you went to work the right way to get it for them. Before condemning, let the English people remember the difficulties the Maidstone committee had in trying to supply suitable treatment during the epidemic of typhoid, notwithstanding their easy access to London, the unlimited number of nurses practically on the spot, unlimited milk supply, &c., and, though I cannot remember numbers, I do not think that their cases were ever numbered by the thousand, as ours are.

---

### Nursing at a Newcastle Stationary Hospital.

By a Sister in Camp.

We are on the point of moving up from Natal into the Transvaal to a new field. Standerton is mentioned as our destination; it is a moderately nice little place, typically Dutch, with a few large stores and some comfortable houses, but, alas! there will be no trees, and no supply of sweet clear water, which we have so thoroughly appreciated here. Our camp, once so full, has now diminished in patients considerably, so that many of our sisters are having a few days' rest prior to renewing their work among our Tommies.

### Pneumonia Cases.

Enteric fever seems to have had its day, and we now receive numbers of pneumonia cases, and some of them are desperately bad too. The climate of Natal is, however, favourable to this disease, and most of such patients fight their way out of it manfully and speedily. We recently had four Boer patients in camp for a short time. It was most amusing to see Tommy's attitude towards his fallen foes. He even went to the length, in some instances, of sharing his soda water and tobacco with them, and many a long talk and joke has passed from British and Boer lip of the day when the Tugela was contended, and when Spion Kop was furiously contested and triumphantly seized. One sergeant "of ours" questioned and cross-questioned his adversaries so tenaciously on every point connected with these battles that it is a wonder the latter did not turn savage; but the Boer, if decently treated, is a wonderfully long-suffering creature, and these—in more senses than one—*patient*-prisoners evinced the customary amount of toleration. They were even photographed two [illegible] three times successively.

### Climatic Conditions.

The weather here has been very variable lately; the most perfectly placid tropical sunshine is often, without any provocation, tempestuously torn asunder by blasts which Boreas himself might have set loose, and then how the tents flap and wrestle with their moorings! And how the tan-coloured dust scuds and scurries past leaving English eyes and mouths too full to cast an ejaculatory blessing on the fugitive. We are under such a blast at the present moment; the ceilings are creaking, and the house is shaking, our candles are blown breathlessly hither and thither, the little yellow flames gasping for life; the noise is terrific. We hear that Boers are within four miles of us, and that our pickets have twice been driven in. What a joke it would be if we were surrounded some night and found ourselves prisoners until help could arrive from either Ladysmith or Volksrust! Some of our party have recently made the ascent of Amajuba. They usually say on returning that they "are very glad they went, although they would not like to repeat the performance!" It is a most formidable undertaking, and demands determination and courage.

### A Dust Storm.

We had a dust storm which lasted the whole day yesterday. Some tents and a marquee came down, and the sisters lived in hourly dread that their swaying shelters would be levelled. They decided to sleep dressed for fear of this happening. No one who has not seen a dust storm out here can imagine the havoc it creates in a tent. To begin with every single thing one has is covered up, swathed in dust, and totally unrecognisable. Cloaks, hats, shawls, boots, basins, jugs, all look as though a rush of lava had swept them and had left them encrusted and unusable. To try and clean things up makes matters ten times worse; patience is the only resource possible under the conditions. "Wait till the dust demon has swept by," and then begin operations—a spring cleaning is not in it! Our poor patients yesterday! The clouds of dust covered them, and, as luck would have it, our water supply was suspended, and we were completely helpless. In the evening, when the storm abated somewhat, we washed our poor Tommies; some of them looked like Arabs, and others were black as Kaffirs!

### A Tea Party.

"No. 14" arrived lately, and this afternoon we are having a tea of dimensions! the whole of both staffs, *i.e.*, medical officers and sisters. Our small front strip of garden, already encumbered with numerous tents, is to be the scene of action. I am wondering how we shall all pack in comfortably, but tolerance and good-humour will come to the rescue, and all will go well. With so sunny-tempered and cheerful a superintendent as Sister Russell everything must pass off successfully; there will be no "angles" possible.

### The End in View.

The night before last heavy cannon at half-past one a.m. caused some little stir in camp. The whole of the men in the "Rest Camp" were turned out, and everyone was wondering what it all meant. The morning brought us tidings that the Boers had blown up the railway lines in two or three places between this and Volksrust, and that our guns had driven them off. This snapshot sort of fighting keeps things lively for officers and for men, as may be imagined, but it is by no means formidable, and the end cannot now be far off.

### Some Hospitals in the Netherlands-Indies.

### III.—THE HOSPITAL FOR THE INSANE AT BUITENZORG.

By a Nurse on Her Holiday.

Nestled amid the temperate shade of glorious waringin trees, and fanned by cooling breezes from the slopes of the now slumbering volcano of Salak, the Dutch East Indian Government Hospital for the Insane at Buitenzorg affords to its five hundred inmates the fullest possible measure of that contentment which, but for their mental instability, they have nothing to do beside enjoy. Under the enlightened direction of Dr. Hofman, aided by the energetic support of his collaborateurs, this institution now justly claims to be carried on upon the most humane, the most rational, and the most up-to-date system for dealing with insane patients which has been brought into existence in any country. It is, in fact, modelled from models, its directeur having visited British India, Europe, and America for the express purpose of collecting and comparing information about asylums and asylum treatment. To this gentleman, and to Dr. Hutshoff Pol, who courteously escorted me over every portion of the hospital, my thanks are liberally due for the particulars I am able to place before the readers of the "Nursing Mirror."

### The Buildings.

The hospital, which accommodates about five hundred persons, is primarily laid out in a males' side and a females' side. Each section possesses separate quarters for first-class cases, second-class cases, and native cases; but first-class ones, and, in fact, all European patients for whom funds are available, are sent to Europe as soon as practicable, it being a recognised principle by the authorities that the climate of Java, albeit cool in the mountains and pleasant enough for those who stay there at will, renders the detention of insane persons adverse to their prospects of recovery, besides being inexpedient on other grounds. The buildings are, for the most part, constructed of brick and plaster, with roofs of highly picturesque red tiles; but those more lately erected are framed in the teak of the country and panelled with a sort of rigid matting made of split bamboo very neatly and closely plaited, which after being set in position is several times coated with lime wash, and provides a cheap, cool, clean, and artistic material for walls, as well as one which does not readily assimilate septic nastinesses withal, and can be readily and inexpensively removed, burned, and renewed if necessary. All the floors are concreted excepting those of the surgical infirmary wards, which are of hard wood, and of the operating theatre, which is of Portland cement dressed on the surface with a non-absorbent glaze. The rooms are amply provided with window space, to which wooden shutters are fitted, glass being wisely dispensed with in this mild and equable climate. The operating theatre is fitted in the best fashion, being in no respect behind any in the military hospitals of the Colony—even those at Magelang and Tjimahi—and much superior to most. Aseptic surgery is the order of the day; and the name of Lord Lister is a household word.

### The Grounds.

The exercise grounds are extensive, level, and laid down in turf; well shaded with waringin trees (a peculiarly handsome variety of Indian fig or banyan), whose delicate foliage is not so dense as to cause the ground to remain long damp, even in the wet season, while sufficiently breaking up the vigour of the tropical sunshine to afford comfort and safety to the patients and nurses. Only the airing courts for specially restive cases are walled round, the others being very lightly fenced. There is no confining wall or boundary trench round the asylum premises as a whole; indeed, a portion of the grounds is occupied by a model farm to which the milder cases among the men patients repair daily with scarcely any restraint or active supervision, and in which they grow the Liberian coffee, maize, rice, kladi (the same as the South Sea taro), cassava, other vegetables, and fruit for consumption in the hospital, and where they rear cattle for milk and beef. No mutton is used; it is not a Dutch food, as sheep do not thrive in Holland, nor do the Malays or Javanese care for it.

### How the Patients are Employed.

The patients are kept employed as much as possible, according to their forms of mental disease and to their previous occupations and modes of life. I noticed several shoemaking, others tailoring, and some at the looms weaving native cloth from cotton grown in the country; there were also carpenters, cabinet makers, and wheelwrights building carts for use in the operations of husbandry undertaken at the asylum farm. There is a small forge, where a good deal, but not all, of the ironwork is made, and where minor blacksmithing repairs are "neatly executed." The patients may earn money, which is put into a box and utilised at the discretion of the authorities, but always for the patients' personal advantage. Some receive small sums for pocket money, with which they are allowed to make purchases in the town on market days. Others save up their dues and go out, when sufficiently recovered, with decent little sums in hand to start life afresh with.

Amusements.

There is a capital little theatre in the hospital grounds for musical and dramatic performances, it being a special and separate building which was constructed and fitted up entirely by the patients, aided out of the unattached funds in the aforementioned money-box for necessary purchases. I was much struck by the neat drop-scene, which was designed and skilfully painted by a patient. Both patients and nurses take part in the representations, and there is a patients' orchestra, or rather band, the instruments of which are provided out of the same source of ways and means.

The Kitchens.

Not far from the theatre are the kitchens, and beyond the servants' quarters and the farm dairy. The kitchens are fitted with steam-jacketted cauldrons, having perforated bottoms and separate but closely-fitting lids. Rice, first soaked in cold water, is cooked in these without the addition of more water save what condenses out of the steam. A meal was just cooked at the moment of my visit to these purlieus, and was being served. I examined it critically, and it seemed in all respects to what may be expected in a good middle-class hotel, without actual luxury. The patients who work on the farm and in the dairy reside in a separate kampong or village, which is not walled or fenced in any way. They are free to wander anywhere if they were so minded; but, as a matter of fact, they remain always on the premises when not engaged in the fields or plantations, feeling that they are better off where they are than they could hope to be anywhere else. They are, of course, selected with great care and caution, but moral discipline is the only kind of restraint exercised over them.

The Matron and Nurses.

The nurses on the female side consist of three classes, and are presided over by a matron, who in appearance is a strong contrast to the more trim and professional-looking sisters. The latter, who are addressed as "Züster," are women of European parentage, and trained in Holland. They wear a neat bluey-grey zephyr uniform, with linen aprons and collars similar to our English nurses, but neither cuffs nor caps, and presented a tidy and clean appearance. Their grade is distinguished by a white or black enamelled brooch in the figure of a Geneva cross, according to the examination they have passed. Dr. Hofman is now training half-caste Javanese girls of good social position as nurses for the women's side, and has begun to meet with encouraging success. Dr. Hutshoff Pol mentioned that he finds he can place much reliance in their intelligence and discretion, and that they are in some respects more biddable than the European Züsters. I saw a pair of these chestnut-eyed and olive-skinned maidens administer a meal by the nasal catheter to a refractory old Dutch vrouw, with gentleness, efficiency, and expedition; they seemed, indeed, to exercise quite a phenomenal power of persuasion, without force of any kind. The third order of nurse is called a Babu, a term of Hindu rather than Javanese or Malay origin, though somewhat altered in meaning by implantation on a foreign soil. She is in reality a "native warder," and acts chiefly as an assistant to the others in watching and controlling both native and European female patients. They do useful service, and the Babus with whom I came in contact seemed to find no difficulty in restraining even the most erratic cases, whom they kept continuously lying upon their beds, the Züster being always within call both by day and by night.

No Padded Rooms nor Strait Waistcoats.

There is not a solitary cell, nor a padded room, nor a strait waistcoat, nor a pair of mufflers, nor any kind of apparatus for mechanical restraint on the premises; the physical persuasion necessary in the most restive cases consists in repressing the patient from rising into a sitting posture on his bed, no matter how persistently he attempts to get up. Sometimes two, or even three, babus, or male attendants, as the case may be, become necessary to effect this; but the method is always the same, and the results are most satisfactory. Epileptics are provided for in a separate room in each section of the hospital. Their mattresses are stuffed with kapok and are placed upon the floor. In all other parts of the hospital the bedsteads are of the same pattern as those in the military hospitals of Java, and are made in the colony. They struck me as the one point open to adverse criticism in this hospital for the insane, being constructed of rather thin iron rod and angle iron—so thin as to need struts and braces of the same material in parts which appeared to me a good deal in the way, and liable to give rise to complications and perhaps injuries in the cases of obstreperous patients. The mattresses are of the "cane-seated" pattern on a teak frame, and answer admirably. There is a post-mortem room in a retired corner of the grounds, and a well-fitted pathological laboratory adjoining the offices, not far from the entrance gate. Finally, after a charming and instructive *tour d'inspection* which occupied an entire forenoon, I came away from this model institution not without many regrets that I was unlikely ever to see it again; and, leaving Buitenzorg on the following day, I proceeded onwards to visit the new military hospital at Tjimahi, in the heart of that part of the country known as the Preanger.

### Appointments.

Taunton Isolation Hospital.—Miss Sarah Armson has been appointed Matron. She was trained at the City Hospital, Birmingham, for two years, and the Coventry and Warwickshire General Hospital for three years. She has since held the post of charge nurse at the General Hospital, Hartlepool, and nurse-matron at the Infectious Diseases Hospital, Foleshill, near Coventry.

### Minor Appointments.

City Hospital, Lodge Road, Birmingham.—Miss Alice Dickson has been appointed Ward Sister. She was trained in the Royal Hospital, Belfast, and was afterwards district nurse in the Hulme District Nurses' Home, Manchester, and has been sister of the medical wards, Mercer's Hospital, Dublin, since August, 1899.

Woolwich Union Infirmary.—Miss Emma Gardner has been appointed Head Nurse. She was trained for three and a half years at St. Marylebone Infirmary.—Miss Mary Christina Byrne has been appointed Night Superintendent Nurse. She was trained at St. George's Infirmary, Fulham Road, London.

Isolation Hospital of Rainhill County Asylum.—Miss Hannah Elizabeth Proudman has been appointed Charge Nurse. She was trained at Salford Union and has since been charge nurse at the Abbey Parish Hospital, Paisley, and night superintendent at Selly Oak Union Infirmary.

Bolton Infirmary.—Miss M. J. Thomas has been appointed Night Sister. She was trained at the General and Eye Hospital, Swansea, for three years, and was afterwards staff nurse. She has since, for the last seventeen months, been charge nurse at the Park Hospital, Hither Green.

### To Nurses.

**We invite contributions from any of our readers, and shall be glad to pay for "Notes on News from the Nursing World," or for articles describing nursing experiences, or dealing with any nursing question from an original point of view. The minimum payment for contributions is 5s., but we welcome interesting contributions of a column, or a page, in length. It may be added that notices of entertainments, presentations, and deaths are not paid for, but, of course, we are always glad to receive them. All rejected manuscripts are returned in due course, and all payments for manuscripts used are made as early as possible at the beginning of each quarter.**

### Echoes from the Outside World.

### AN OPEN LETTER TO A HOSPITAL NURSE.

A SECOND dreadful disaster has visited America. On the previous occasion it was fire which consumed the poor people in the vessels like rats in a trap, unable to escape; this time it is water which has been the destroying element. But the last calamity is much worse than the first, for not only has a whole city been destroyed, and £2,000,000 worth of damage been done, but several thousand lives have been lost. The matter, too, touches us nearly at home. Galveston had the largest cotton-exporting trade in America, and Lancashire will suffer greatly from the disaster in Texas. The crop for next year is badly damaged, so that the yield will be much diminished, and there will necessarily be much delay in exportation. All this means bad times for cotton spinners and manufacturers. The force of the inrushing waves may be gauged by the fact that schooners and steam launches, which were in harbour at Galveston, are now ten miles inland.

I HEAR from a correspondent in China that those who have been prisoners in Ti entsin are anxious to show their gratitude to the soldiers and marines who defended, or extricated, them from their deplorable position. Mrs. Scott, wife of the Bishop of North China, is starting the movement in the absence of her husband. He, good man, has returned to Tientsin to render assistance in the work of mercy to the wounded, whilst she is at Wei-hai-Wei. There she is a frequent visitor at the hospital, and she is much impressed with the terrible nature of the wounds of many of the poor fellows, which she fears in several cases will incapacitate them from earning a livelihood even should they emerge from the hospital alive. If the widows and dependent relatives of those who died in the struggle be added to the list of those who have suffered from the Boxer insurrection, it will at once be seen that there is likely to be need for a good deal of money in the future. It is fitting that those who have most cause to rejoice at their rescue from worse than death should show their thankfulness in such a practical manner, and Mrs. Scott, having spent ten days in a cellar under the Gordon Hall at Tientsin, writes feelingly. It is probable that, notwithstanding their recent liberality to the South African War and the Indian Famine Funds, the British residents in China will respond liberally to the appeal.

IT is generally believed that, as soon as peace is formally declared in South Africa, there will be a great exodus from this country of all sorts and conditions of men—and women. I have not heard that there is likely to be a special demand for the services of nurses. But this must, of course, ensue if there is a considerable increase in the population, for, unfortunately, though trade follows the flag, so also, in the natural order of events, must sickness. Happily, we who send the settlers can also supply the doctors and the nurses required to minister to their necessities. At present, so far as women are concerned, the cry appears to be for dairymaids. The Boer women cannot, or do not, make decent butter any more than the natives, but there is ample material for both butter and cheese in the Transvaal, if only there were persons who knew how to make them properly and cleanly. In time the separator may be found useful, but this can be dispensed with until the day comes when the implements employed in farming at home will be exported wholesale. But there are plenty of Kaffirs to hand ready to turn the rough churns, which do their work perfectly well when properly manipulated. Young women who understand poultry and know how to rear chickens as well as how to feed hens for the production of eggs can also find an opening in our new possessions.

I AM glad to see that some of the magistrates of Lancashire are emulating the example of Admiral Field, which I commended the other day, and have warned the holders of public-house licences that unless they provide tea and light refreshments when they are asked for them they must expect to be refused a renewal of their licences. In Lancashire, also, the police officials are interesting themselves in the matter, and one of the superintendents has been the means of bringing several of the offenders to book. Having received a number of complaints from cyclists that they could not obtain light refreshments he very properly investigated them, and having ascertained that they were well-founded brought the matter before the bench. If superintendents elsewhere will only be equally diligent in the discharge of their duties, women cyclists will soon be able to ask for a cup of tea at a public-house with the same confidence that the male sex demand a glass of beer.

BUT the fair cyclist in America, according to recent news, will not only find it impossible to get served with a cup of tea, but with refreshment of any sort, should she be attired in what is called "rational costume." The only thing she is likely to receive is chastisement, and chastisement which, according to "the judge," is so highly deserved that she can obtain no compensation. A man of Washington, Connecticut (naively described as a gentleman), horsewhipped his wife in the open street the other day because she disobeyed his orders, and wore a bloomer costume in public. Her companion, also attired in knickers, who came to her rescue, was likewise severely lashed by the infuriated man. The man was fined £2 in the police court for the assault, but "the judge" at once remitted the money, because, though legally guilty, he considered that the defendant deserved the thanks of society for his courage in resisting a practice, becoming all too common among women of the States, of appearing publicly in a costume not sanctioned by modesty. I wonder how many husbands in England would venture to copy this example, and use brute force upon their wives? I rejoice greatly that the married women of my acquaintance who wear bloomers are extremely limited, or I should be in daily fear of hearing of their decease or serious injury.

WHAT would the American magistrate say to the director of Kew Gardens, who has found himself in a fix, and got out of it in a most original manner? When first the three lady gardeners were engaged to assist in the work at this favourite horticultural resort they were asked what dress they would prefer to wear, and the bloomer costume was suggested. But the result was not successful. The walls of Kew Gardens were not high enough to prevent inquisitive folks staring over, and much annoyance was caused by people getting on the tops of omnibuses to see the ladies at work. Accordingly, the director signified that they must wear a dress "similar to that of the ordinary gardener," and they promptly appeared in suitable costume. Query, what is the dress of an ordinary gardener? Is it the rather shabby dark dress, shady hat, apron and gloves of the lady genus, or the fustian trousers, shirt sleeves, and bowler hat of the male kind? It would be interesting to know.

THE news has been flashed from Lourenco Marques that Mr. Kruger has arrived at that town. This, coupled with the report that General Botha is in favour of surrender, seems to justify the opinion that even the leaders of the Boer rebellion are recognising the inevitable. Meanwhile, "B.-P." has become a policeman—that is to say, he has accepted the highly responsible military position of chief of the Transvaal Police.

### Experiences in a Plague Hospital.

By a Nursing Sister.

(*Continued from page* 308.)

### The Convalescent Wards.

At the beginning of the epidemic, when I first came, we had only four wards; but we have since opened two others for the convalescents, and I am glad to say that the one for the plague patients is always full. The wards hold twenty beds comfortably, but we often have to put several extra up the middle. Patients seem to come in rushes; on some days we have as many as twenty admissions, and hardly any for three or four days. Sometimes five or six ambulances will arrive in quick succession, and the sisters in the bungalow pity those on duty.

### The Night Duty.

There were five sisters here in February, which was our busiest month; two were on duty in the morning, two in the afternoon, and one at night. The night duty is the worst of all; fortunately we only do it for a week at a time, and we find it quite long enough. It is very gruesome work, going through the dimly-lighted wards, often met with the news that "Number So-and-so is dead, sister," or else finding out for yourself that a patient, apparently sleeping, is dead, and the ward servants have not known it. The acute plague ward is always a distressing sight, I think, as the patients are nearly always delirious, and have to be tied down hand and foot to prevent them from getting away. Some will often not sleep for a moment the whole night, and call out and answer each other incessantly.

### Singing in Delirium.

There was a man with a most melodious voice who would suddenly begin to sing at all hours of the day and night. One day the doctor asked him why he made such a noise, and he said, "How will the bullocks go if I don't sing to them?" By the sounds he made he was evidently driving some, and several times he seized my hand and worked it violently up and down, singing lustily. I suppose he thought he had got hold of the bullock's tail, and was trying to drive me. I am sorry to say that he died, quite suddenly, after we had begun to think he would get better. He and his wife had been brought in together, and she had died on the day after admission.

### A Very Sad Case.

We had a sad case of a mother who died and left four children, the eldest a boy of about ten years old, and the youngest a girl of two. The boy had been a most attentive little nurse, soothing and petting his mother as if she were the child. He wept bitterly when she died, and so did the elder girl, and the little ones cried because the others did. The boy seemed to realise at once that he was the bread winner of the family, and told Sister that he was going out to look for work. She let him go, but got permission from the doctor to keep them all for a time, till it could be arranged for one of the sisterhoods to adopt them, which they are always willing to do. A few days later the big girl developed small-pox, and had to be taken to another hospital; so, till she is quite well, we are keeping the others. The baby has a cot in the women's ward, where she is getting fat and spoilt; she is the most fascinating little thing, just beginning to talk, and she has grown wonderfully since we have had her. All the ayahs want to adopt her, but I do not think we shall allow that.

### The Ingratitude of Patients.

When the plague patients are discharged they all receive money, clothes, and a blanket from the officer of the district, who comes several times a week to see if anyone needs help. He also keeps us provided with biscuits and oranges out of the Charity Fund, which is on purpose for giving little luxuries to the patients. What one notices very much with these people is their ingratitude; after being nursed and fed for several weeks perhaps, and finally cured, they will go without a word, not even salaaming if they pass you in the ward. Of course, there are exceptions, and one or two have actually come back to see us afterwards. Also, some of them have come as patients a second and third time, which shows they appreciated the care they received, though they may not have expressed their gratitude in words.

### Parsee Practices.

The Parsees, on the other hand, are very different; there is quite a grand ceremony when one of them is going out. The sisters who are not on duty are asked to come to the ward when the doctor comes round, and then wreaths are placed round everybody's neck, and bouquets are presented, and there is much salaaming and cracking of knuckles. The latter is a curious little custom, and it has a pretty meaning. They quickly stroke you down, from your face to your feet, and then putting their knuckles to their ears, they crack them, saying something, I don't know what, but meaning that they take all your sins and troubles upon themselves, so that you may lead a happy life. They only do this when they are especially grateful for anything; and it was done to me the other day by a little woman, when I had finished her daughter's long and painful dressing, which took me nearly an hour every day and tired me fearfully.

### The Doctor.

Our doctor is a native and a very clever man; he qualified at Edinburgh, and it is his own treatment of plague that is used here. Nursing goes for something, and skilful feeding is a most important thing. A native doctor is more useful than an Englishman, as he knows the language and customs and also understands the various kinds of country fevers. It is not easy to diagnose plague in the first instance as the high temperature may arise from pneumonia or ordinary fevers, and in the absence of swellings you have to watch a case for some time.

### Visit from Lord Curzon.

We had a visit from the Viceroy when he was in Bombay for a few days inspecting all the plague hospitals. A guard of honour, dressed in scarlet with turbans to match, accompanied him, and the state carriage was drawn by four horses, with outriders. Lord Curzon was very nice, and asked me how long I had been out, and if I liked the work. He made many inquiries about the patients, and was interested in the treatment, going all round the wards, and expressing great satisfaction at everything. The doctor was delighted with his Excellency and his evident appreciation of the work done in our hospitals.

### Cases of Recovery.

One little boy with pneumonia calls me "Missie Baba," an inappropriate name for one so tall; he is very fond of me, and the other night he was saying all the time he wanted his "Missie Baba." I asked him where his pigtail was, and he said he cut it off before he came in, because he thought he would be held by it and beaten. We have two good cases of plague recovery in the women's ward. One patient had been delirious and unconscious, and speechless for over a month, and we never thought she could get over it. She used to scream like a peacock whenever she was touched or fed, and the name has stuck to her; she is nearly well now, and so delighted at being able to talk again. The first word she said was "utcha" (better); and if you ask her what name sister calls her, she points to me and says "Peicko," and goes off into fits of laughter. Another little woman just opposite her also lost her speech, and it is very funny to hear her talk, as she can't control her voice at all, and it goes up and down anyhow. Her first word was "kana" (dinner), as she used to cry dreadfully when the rice and stuff were given out to the others, and was always making signs that she wanted food. The two women sit up and talk to each other now, and the "Peacock" keeps the other one in order, and reproves her when necessary. A delirious man escaped in the evening once, and after a long hunt he was found in the sea, just outside the hospital, up to his neck in water, and with difficulty he was brought safe in again. He belonged to one of the ships in the harbour, and his idea was to swim back to it. I gave him some brandy, and he seemed none the worse for his sea bath. Patients often run away, and then the ward servants are fined. One was never found at all; he went off with a blanket and sheet because we wouldn't give him full diet.

A Curious Custom.

We have several children in the hospital, all convalescent, and so nice to play with. A lot of toys have been sent by a friend from England, and the dolls, balls, and picture books are a great delight. One mite of a baby has been busy feeding a doll with biscuits, and enjoying her game. A dear little soldier doll was given to the smallest boy we could find, and he sat and smiled at it in the most amusing way. There is a custom here that women must not say the names of their husbands. The doctor is very fond of asking them and seeing them refuse; he asked a woman the other day, and she would not tell; but the husband himself was there, and told her to. Even then she did not like it, but at last burst out with the name, and promptly retired under the blanket. It was a funny sight! On the whole I find plague work most interesting, and the life here is very pleasant.

### Everybody's Opinion.

[Correspondence on all subjects is invited, but we cannot in any way be responsible for the opinions expressed by our correspondents. No communication can be entertained if the name and address of the correspondent is not given, as a guarantee of good faith but not necessarily for publication, or unless one side of the paper only is written on.]

### COLD BATH-ROOMS.

Dr. Urquhart, of Perth, writes: I suggest that there is a more excellent way of warming bath-rooms than your correspondent has indicated in The Hospital for this week. The kitchen fire must be kept going, and the hot water for domestic purposes ought to be plentiful at all times. Now, much of the fuel used in the kitchen is not taken advantage of if it merely escapes up the chimney after heating a boiler only connected with a cistern. Some years ago I introduced into my bath-room a coil of copper pipes opening at either end into the rising main from the boiler, for the purpose of drying towels and keeping the room at a reasonable temperature. It proved so effective that I formed another coil under the window of my library, which is a larger room than can be properly heated by an open fire. Consequently, a smaller fireplace was found sufficient, and yet the kitchen boiler is not overtaxed by the extra work demanded of it. The cost of warming a bath-room as thus suggested is merely the cost of the apparatus, and this method is much to be preferred to lamps on the grounds of safety, hygiene, and economy. Perhaps I ought to add that the kitchen boiler is fitted with a dead-weight safety valve in an accessible position, although we have had no trouble with frost since the pipes were properly placed and protected by a non-conducting cement made of clay and jute waste.

### NURSES AND MENIAL WORK.

"Nurse B." writes: Will you allow me to make a few remarks on this subject? I am a constant subscriber to The Hospital, and have often wondered that this subject has not drawn a wider discussion among nurses. In many hospitals the charwoman business gets much more attention than the nursing, I fear, for some inexplicable reason on the part of the authorities. I am so much accustomed to hear that "nothing ought to be beneath the dignity of a nurse." The observation is generally made by those who have only to superintend. But can one make it in good faith? Dignity, which ought to be a nurse's aim in discharge of duty, lies far apart from scrubbing, polishing, sweeping, &c. Any being of ordinary cleanly habits would know how to get dirt removed without this vigilant training; but after a time in hospital, would they know how to nurse or superintend nursing? How much depends very often on the work of the nurse! It is not her excellence in scrubbing that saves her patient's life. And this severe manual labour, in conjunction with the mental strain of nursing, very often breaks up the constitutions of healthy women, and then—who wants them? But the servants' work of institutions is thus done cheaply.

### OUR DEALINGS WITH THE DEAD.

"Nurse M." writes: Seeing this article in The Hospital it recalled to my mind the most unsanitary custom prevalent amongst the poor of leaving the dead exposed in an open coffin until the day of the burial. Where the body is in good condition this signifies no danger to the near public, but where the body has been in a state of decomposition it must be positively dangerous. The case which came especially under my notice was that of a poor woman who was "laid in state" for a week in the tiny parlour of a tiny house. The children were permitted to run in and out "to have a look at poor mother to cheer them up a bit," as the aunt remarked, and all who came to the house were cordially invited to look at the corpse, already gruesomely changed. The presence of that body was strongly manifest the minute I entered the street door, and the relative was most incensed and hurt when I refused to look at it and told her how foolish it was to disobey the undertaker, who had remarked that the coffin should be closed as soon as possible. I tried to persuade her to have it done then, but she refused, thinking me no doubt a callous wretch, and the corpse was finally buried in a high state of decomposition on the ninth day after death. That the closing of the coffin should be left to the discretion of a set of ignorant and sentimental friends or relatives is a scandal which can only be put a stop to by law. In Germany, three days is the limit for the body to lie in state, after which the relatives are forced by law to have the coffin closed. So it should be here.

### NURSING IN MILITARY HOSPITALS.

"A Sister of the A.N.S.R." writes from Netley: There is much indignation felt here by the Army Nursing Sisters and sisters of the A.N.S.R. at an article which appeared in your issue of August 25th. We conclude it has been written by a sister just embarked for South Africa, who had not had the courage to express her opinions and remain here. The article is in the worst possible taste from every point of view; a few jesting remarks construed into honest opinions, the writer herself being the only one who grumbled at "the length of the corridors," "the early rising," or "need of extra time off duty," thereby willing to leave her patients for longer time in charge of the much-abused (by her) orderlies. Your Netley correspondent lives to reorganise, and "would wish to make crooked things straight which were meant to be crooked." I have been at Netley for the past seven months, and have the highest appreciation of the manner in which the hospital is worked under the supervision of Miss Norman, lady superintendent, who ranks first in the nursing profession. A member of the A.N.S.R., it is my ambition to join the Army Nursing Service. The sisters here, both permanent and temporary, have worked on the best of terms with the members of the R.A.M.C., and it is not our intention that the friendly feeling which exists between sister and orderly should be destroyed. They do all the disagreeable part of nursing, while ours is the more interesting and refined. Yet they are always willing to help us, cheerful and obliging. It would indeed be a pity if the opinion of an individual, herself unappreciated by patients, orderlies, or sisters, should be taken seriously as a general sentiment. If your correspondent thinks so badly of the military system of nursing, and has failed to improve it, why does she remain member of a service to which she cannot be loyal and yet criticise so severely?

### A Book and its Story.

### THE COUNTRY OF THE HOUR.

Among the various works dealing with China, and the Chinese, "unfathomable, unregenerate, among the races of the earth," none more exhaustive and interesting has appeared than that of Eliza R. Scidmore's.* The authoress has had fifteen years' intimate experience of the country, and is therefore competent to speak with authority, based on actual intelligent observation, of its customs and national characteristics. And yet, in spite of this, she confesses that during the seven return visits paid, each one but increased the mystery of its people and the enigma of the future.

"No one will ever know the Chinese—the heart and soul and springs of thought of the most incomprehensible, inscrutable, contradictory, logical, and illogical people on the earth. Of all Orientals no race is so alien. Not a memory, not a custom, not a tradition nor an idea, not a root-word nor a symbol of any kind associates our past with their past. There is little sympathy, no kinship nor common feeling, and never any affection possible between the Anglo-Saxon and the Chinese. Nothing in the Chinese character or traits appeals warmly to our hearts or imagination, nothing *touches*; and of all the people of earth they most entirely lack 'soul' charm, magnetism, attractiveness. We may yield them some admiration on intellectual grounds, but no warmer pulse beats for them. They are chiefly points of contradiction between them and ourselves."

On the monotonous, paralysing effect of sameness, in physical appearance, dress, and mental mould; on the vastness of the race, with its prejudices, superstitions, and childish customs; its wearying repetition of life, character, and incident, she writes, "The same yellow skin, hard features, and harsh mechanical voices; the same houses, graves; the same selfish conservatism, blind worship of precedent and antiquity, that offend one almost to resentment." Naturally, to an American, which the authoress is, these national traits would be peculiarly antipathetic. "Without patriotism, decadent, indifferent as to who rules or usurps the throne, opposing all who would alter the existing state of things, there is yet no country in which political martyrs have met death more nobly than those reformers who were executed at Pekin so lately as 1898." There are optimists who see in the Chinese "the world's greatest and earliest teachers, its future leaders and rulers, its chosen people; . . . destined to outlive, override, and outdo all the pale races; the whole hope of humanity bound up in this yellow people." This seems difficult of belief, impossible to realise, unless the Chinaman becomes a different being. The effects of countless generations, in which corruption and cupidity, with their attendant evils, have held sway is seen in the present condition of the country. "There seems no living spring nor beating heart in the inert mass; their three great religions are dead; two systems of ignoble superstition live. Literature is a fossil thing; the arts have died; the genius of the race has fled." Their one great virtue—filial piety—has become degraded into the cult of ancestor-worship; women are held in no respect, and regarded as inferior creatures. And yet, true to its enigmatical character, China has allowed its throne to be usurped three times within the last forty years by a woman, and to be hurried to its ruin in consequence. The Empress-Dowager, as all the world knows, is a woman of no ordinary parts. In appearance she is said to be "tall, distinguished, with pronounced Tartar features, eagle eyes, and a particularly penetrating voice." This portrait, an early one perhaps, in no way disagrees with the later one, describing her as a high-tempered, vindictive, old Manchu dowager odalisk. The shrewdest woman in Asia, the "only man in China," and the most unbridled female despot the world has known. She rose from the harem's rank, uneducated, ignorant of public affairs, but by her strength of mind and dominant personality, her wits and duplicity, she has attained supreme power.

With the plots and counter-plots which have edged the Emperor, her nephew, everyone is more or less familiar. "In all topsy-turveydom surely nothing approaches this petticoat tyranny and bullying of the puppet Emperor, poor Kwangsu. The one man in palaces, full of women and eunuchs, yet unable to free or assert himself; a mannikin majesty who is put off and on the throne at a short notice; set up and lifted down, like a marionette or a piece of furniture, without as much as 'By your leave.' A pitiful 'Paper Tiger' of an Emperor."

The authoress writes of Pei-ching (Peking), the northern capital, the city of universal interest at this moment as, "Two cities, the Chinese and the Tartar City. The outer and the inner city lie side by side, each entirely surrounded by a great defensive wall, the Manchus' citadel even more strongly walled and defended from the Chinese quarter than the outer plain. The Tartar or inner city, as it is called, holds in its centre the Yellow or Imperial City, and within that again is the Purple Forbidden City, the actual palace enclosure, the home of the Son of Heaven. . . . One first enters the Chinese City through a deep arch in the solid walls, and after two miles comes to the more impressive walls and gate towers of the Tartar City. Legation Street is reached through the deep arch of the Hatamen. All the foreign compounds are in or near the street, a straggling unpaved slum through which an occasional European may be seen picking his way between ruts and puddles with the donkeys and camels. Envoys, plenipotentiaries, and scions of *la carrière diplomatique* having lived along this broad gutter for nearly forty years, have had just as much effect upon Imperial Pekin as many barbarians had upon Imperial Rome."

Mission work in China has an interesting chapter to itself. As early as the seventh century Rome sent a papal embassy to China, and Nestorian Christians had already been proselytising for a hundred years. From that century to the present Christian missionaries have been zealously at work. To the early Jesuit fathers the conversion of the reigning house of that date is due. And their intellectual influence was supreme. They were in later centuries, notably the seventeenth, actively instrumental in directing the artists in the palace ateliers; designing and decorating the royal villas, producing new colours for the native potters, and founding glass works by the northern cathedral, from whence many beautiful works of art came. "After 1860 only, Protestant missionaries, availing themselves of a surreptitious clause in the French treaty of that year," became established in Peking. The visible statistical results of Christian missions to-day shows one million Roman Catholics and 50,000 Protestants. The social, no less than the historical, aspect holds the reader's attention. An audience, desired by a Chinese noblewoman of our authoress, gives a glimpse of the costume and interior life of the Manchus of rank: "After following the guide through spacious flagged courts, where lattice-windowed dwelling-rooms lined each side, we reached a noble ancestral hall with green-tiled roof and tile-lined walls, massive red lacquer columns supported the roof, itself falling into decay, but shining out—a gorgeous mass of colour against a brilliant sky." A dazzling group of ladies awaited their guest. "Each one, more brightly picturesque than the other, was lifted upon stilt or flower-pot shoes. Their robes were of the richest brocades and satin, in varying shades of softest tones, embroidered in silk and gold." It is not possible to do more than present to our readers in the space permitted some points of interest, in a work like the present, dealing with so vast a subject; but we hope that enough has been said to excite interest in it.

* "China the Long-lived Empire." By Eliza R. Scidmore. (Publishers: Macmillan, London. One vol.)

### For Reading to the Sick.

A friend loveth at all times, and a brother is born for adversity.—*Prov.* xvii. 7.

Souls that carry on a blest exchange
Of joys they meet with in their heavenly range,
And, with a fearless confidence, make known
The sorrows Sympathy esteems its own—
Daily derive increasing light and force
From such communion in their pleasant course,
Feel less the journey's roughness, and its length,
Meet their opposers with united strength,
And one in heart, in interest, and design,
Gird up each other to the race divine. —*Cowper.*

**Reading.**

Whoso feareth the Lord shall direct his Friendship aright; for as he is, so shall his neighbour be also.—*Ecclus.* vi. 17.

Love everyone with the pure love of charity, but have no friendship save with those whose intercourse is good and true, and the purer the bond which unites you so much higher will your friendship be. Your intercourse is praiseworthy if it arises from a participation in goodness, prudence, justice, and the like; but if the bond of your mutual liking be charity, devotion, and Christian perfection, God knows how very precious a friendship it is. Precious because it comes from God, because it tends to God, because God is the link that binds you, because it will last for ever in Him. Truly it is a blessed thing to love on earth as we hope to love in Heaven, and to begin that friendship here which is to endure for ever there. I am not now speaking of simple charity, a love due to all mankind, but of that spiritual friendship which binds souls together, leading them to share devotions and spiritual interests, so as to have but one mind between them. Such as these may well cry out, "Behold how good and joyful a thing it is, brethren, to dwell together in unity!" Even so, for the "precious ointment" of devotion trickles continually from one heart to the other, so that truly we may say that to such friendship the Lord promises His Blessing and life for evermore. To my mind all other friendship is but as a shadow with respect to this, its links mere fragile glass compared to the golden bond of true devotion. Just as men traversing a plain have no need to hold one another up, as they have who are amid slippery mountain paths, so those who are living in the world require such for strength and comfort amid the difficulties which beset them. We cannot bestow friendship on many persons; the highest grace consists in having no friendships but those which are holy, good, and true.—*Francis de Sales.*

A love that gives and takes—that seeth faults
Not with flaw-seeking eyes like needle-points,
But loving-kindly ever looks them down
With the o'ercoming faith of meek forgiveness!
—*Lowell.*

Can we forget one Friend? can we forget one face
Which cheered us toward our end, which nerved us
for our race?
Oh! sad to toil and yet forego
One presence which has made us know
To God-like souls how deep our debt!
We would not—if we could—forget! —*Kingsley.*

### Wants and Workers.

Will anyone having a water bed or pillow for disposal, let an invalid have it for a small sum? Address Mrs. Payne, Brynkmalt Cottage, Chirk, North Wales.

### Notes and Queries.

**The Editor is always willing to answer in this column, without any fee, all reasonable questions, as soon as possible.**
**But the following rules must be carefully observed:—**
**1. Every communication must be accompanied by the name and address of the writer.**
**2. The question must always bear upon nursing, directly or indirectly.**
**If an answer is required by letter a fee of half-a-crown must be enclosed with the note containing the inquiry.**

*Weir-Mitchell.*

(238) I have been ordered the Weir-Mitchell treatment for nerve depression. Can you recommend me a home or hospital, in a mild locality, where the terms are moderate?—*Anxious.*

See advertisements in the "Mirror" for private homes.

*Nursing Home.*

(239) Is there a directory of private nursing homes in London? I have had some training in a cottage hospital, and wish to go to Egypt in the winter. I am willing to pay a premium, as I have only time for a short training, and I am not strong enough for a large hospital.—*Difficulty.*

There is no directory of nursing homes, neither is there any means of discovering them, excepting by means of the Post Office Directory. Nurses cannot be made by a short training.

*Mental Work.*

(240) Having had some nine years' experience in mental nursing without training, do you think it possible for me to get employment in an asylum, London preferred? I am over 40, and in good health.—*Mental.*

You might apply to the Secretary, Metropolitan Asylums Board, Victoria Embankment, W.C. Assistant nurses, Class II., do not require training, and are eligible for promotion to Class I. Wages begin at £20 a year, and increase annually.

*Consumptive.*

(241) Can you tell me of any convalescent home where a consumptive girl, not bed-ridden, can be taken in for a few weeks' change? Ventnor Hospital is too expensive.—*L. M. C.*

All Saints' Convalescent Home, 8, Pevensy Road, St. Leonards, terms (without letter) 7s. 6d. a week, might be suitable. See "Burdett's Hospitals and Charities" for list of convalescent homes.

*House-fellow.*

(242) I am most anxious to meet with a companion—a retired nurse—to share my country cottage. I would give her accommodation, if she would furnish her own bedroom and pay her share of living expenses.—*District Nurse.*

This kind of arrangement might be made very advantageous to many nurses, providing, as it promises, companionship to the working, and a home for the superannuated nurse. The difficulty of selecting congenial house-fellows is very great, however.

*Brazil and Chili.*

(243) Can you tell me of any hospitals where English nurses are employed in the West Indies, Brazil, or China? Also to whom to apply—*Ward.*

The General Hospital, Barbados, Miss Caroline Walker; San Fernando Hospital, Trinidad, Mrs. G. C. Fidler; Anglo-American Hospital, Rio de Janeiro, the Matron, are the most important.

*Probationer.*

(244) Do you know of a surgical or children's hospital within 30 miles of Brighton where a probationer of 18 would be trained without premium?—*R. C.*

Twenty is the lowest age at which probationers are received in any of the Brighton hospitals.

*Travellers' Aid Society.*

(245) Will you kindly tell me in your "Notes and Queries" column if there is a branch of the Girls' Friendly Society or the Travellers' Aid in Paris; and, if so, would they send someone to meet a girl at one station, and take her across Paris to another, and arrange about her ticket, registration of luggage, &c., as she does not speak French?—*A Subscriber.*

If "A Subscriber" will write to the secretary of the Travellers' Aid Society, 3, Baker Street, London, W., giving full particulars, and three days' notice, arrangements can be made for her to be met in Paris, and taken from one station to another.

*Outside Lectures.*

(246) I should be glad to know which of the London hospitals admit outsiders to the lectures given to the nurses.—*J. G. C.*

This custom is not practised by any of the more important training schools. There are other agencies providing nursing instruction for amateurs: St John Ambulance, The Polytechnic Institutes, and the Young Women's Christian Association.

**Standard Books of Reference.**

"The Nursing Profession: How and Where to Train." 2s. net; post free, 2s. 4d.
"The Nurses' Dictionary of Medical Terms." 2s.
"Burdett's Series of Nursing Text-Books." 1s. each.
"A Handbook for Nurses." (Illustrated.) 5s.
"Nursing: Its Theory and Practice." New Edition. 3s. 6d.
"Helps in Sickness and to Health." Fifteenth Thousand. 5s.
"The Physiological Feeding of Infants." 1s.
"The Physiological Nursery Chart." 1s., post free 1s. 3d.

All these are published by The Scientific Press, Ltd., and may be obtained through any bookseller or direct from the publishers, 28 & 29, Southampton Street, London, W.C.

### Travel Notes.

### LVII.—A FORTNIGHT IN SWITZERLAND.

TO-DAY I am going to tell you how to spend a fortnight in Lucerne for the modest sum of £9. The journey, from its length, is unavoidably expensive, but once there a good managing traveller may live for even less than the sum named. Dr. Lunn advertises some remarkably cheap trips, and for the nervous and inexperienced they are excellent, and offer advantages in the way of good accommodation and a small expenditure of money that is indeed surprising. I unhesitatingly recommend them to all who do not feel able to battle for themselves, and I know from personal testimony that they give great satisfaction. There is, however, one drawback. You are always kept moving from place to place, which is a great weariness to those who want a real rest in every sense of the word. It is for such that I shall sketch a plan for a holiday in Lucerne and other places in Switzerland where living is cheap and peace and quiet may be enjoyed by the overworked.

### THE JOURNEY ITSELF.

The best way is by Dover, Ostend, Brussels, and Strassburg; cost, second-class return, £5 7s. 6d. This ticket lasts for forty-five days, so that if time and money admit of so doing you may remain that length of time on the Continent. There is a cheaper route by Newhaven and Dieppe; cost, second-class return, £5 0s. 10d., but it only allows of a trip of nine days, and personally I think it ridiculous to take so long a journey to spend only six days stationary. The Polytechnic has a cheap trip, "A week in lovely Lucerne for £5," but think of the toil. Going by Dover and Ostend the journey is not at all fatiguing. You leave London at 9 a.m., and have a daylight crossing, reach Brussels about 6 p.m., where there is twenty minutes' halt. Strassburg is reached at four in the morning, but only a stop of five minutes is allowed. At Bale there is always a good hot breakfast ready of coffee and rolls, and all advantages for a wash and brush up. Lucerne is entered at 9 a.m. So you see it is only twenty-four hours, and is really an easy journey, not too much for anyone in ordinary health.

### LIVING IN LUCERNE.

Accommodation may be had in Lucerne to suit all purses. Anyone wishing for addresses has only to apply to me, and I shall be happy to help them; but space will not allow me to enter into details here. If those applying to me for advice as to hotels would in all cases give me a hint of what they wish to spend per week it would simplify matters greatly, and I should be able to help them much more effectually.

### THE TOWN ITSELF.

The situation of Lucerne is ideal, and the way in which the old town lies along the edge of the lake is fascinating. All the quaint buildings and arcaded streets are reflected in the water, and nothing could be more picturesque than its general effect. The two bridges, called the old and the new—though, as a matter of fact, both are old—are very striking. Their proper names are the Kapellbrücke and the Spreuerbrücke. Of the two the Kapellbrücke is the more quaint, from the old Wasserthurm which adjoins it in the middle of the rushing Reuss, which is usually of a bright green colour, an effect generally observable in rivers that flow through the snow mountains. There is very little art or architecture of any note in Lucerne. It is not in those things that its charm lies.

### THE HOFKIRCHE.

This old church is remarkable for its magnificent organ, which is played from half-past six to half-past seven every evening in the season. There is also a fine carved pulpit and beautiful stalls of the sixteenth century. Immediately behind the church is the old churchyard, enclosed by arcades curiously frescoed. The church and churchyard are raised high above the town, and you mount to them by a flight of steps at the end of the main promenade by the lake.

### THE CELEBRATED LION.

This is to be seen free at all hours, and is a splendid piece of work. It commemorates the heroic stand made by the Swiss Guards in defence of the Tuilleries in 1792. The lion is hewn in the solid rock, and is represented as being transfixed by a lance broken short off; under his paw is the Lily of France. It is a great pity that it is now surrounded by tawdry booths and an odious panorama.

### ASCENT OF THE RIGI.

This is the first excursion which everyone is anxious to make, and now that one ascends prosaically, but easily, by the railway, it is within the reach even of the feeblest. The line is managed on the rack and pinion system, and is as safe as one's own drawing-room. From Lucerne the ascent should be made from Vitznau; the steamer lands you at the little quay, and the train starts from it. The best plan is to go thus (fare seven frs.), and to return on foot via Weggis, where one picks up the return boat. Start early in the morning so as to have a long time to wander about the summit before returning in the evening. If you are able to stay a couple of nights on the top of the Rigi so much the better, because the walks are endless round and about the summit. But do not be rash—mountain exploring is rather a dangerous proceeding if you forsake the well-worn paths. A very delightful day may be spent by going up the Rigi line to Kaltbad, and then turning off to the Rigi Scheidegg, four and a quarter miles (also by train). From here there are some good walks, but I do not consider them fit for inexperienced mountaineers. A good walk—three and a half hours—will take you from Gersau, across the lake to the Scheidegg, and there are no difficulties to be encountered; but remember that three and a half hours up will certainly mean two and a quarter down. Next week I shall speak of Pilatus and the lake excursions.

---

### TRAVEL NOTES AND QUERIES.

RULES IN REGARD TO CORRESPONDENCE FOR THIS SECTION.—All questioners must use a pseudonym for publication, but the communication must also bear the writer's own name and address as well, which will be regarded as confidential. All such communications to be addressed "Travel Editor, 'Nursing Mirror,' 28, Southampton Street, Strand." No charge will be made for inserting and answering questions in the inquiry column, and all will be answered in rotation as space permits. If an answer by letter is required, a stamped and addressed envelope must be enclosed, together with 2s. 6d., which fee will be devoted to the objects of the "'Hospital' Convalescent Fund." Any inquiries reaching the office after Monday cannot be answered in "The Mirror" of the current week.

KNOKKE (Bonnie Scotland).—I do not think you will meet many English, it is such a very quiet little place, and it is getting late in the season. I hardly think you would enjoy it if you cannot speak French, unless you are going with a companion. The lack of French would not matter much, I think, from a business point of view. Write to the proprietor of the Hotel de Bruges, and ask him his terms *en pension*. You must remember that your journey would be much more expensive from Lille than from England by water. If you do not mind quiet, and have an agreeable companion, I think you will enjoy the place.

KNOKKE (Mirror).—As you will see, questions on Knokke are numerous this week. See answer to "Bonnie Scotland." The journey from Tower Bridge takes about 18 hours, but it varies considerably. A good passage is much under that time.

KNOKKE (Northern Lassie).—Quite possible to do it on the lower sum you mention and without pinching. French is spoken. See answers to "Mirror" and "Bonnie Scotland."

THE RIVIERA (Sapphire).—No, it is not altogether an ideal country for cycling, because there are comparatively few high roads. If you want to visit the mountain villages lying to the north, some can be reached by cycling, but most only on foot or with a mule. At the same time, with a combination of the two, if you are a good pedestrian, everything may be seen. Be sure to have a good brake on your machine; there are some very dangerous places even on the Corniche.

PAU (Despair).—If you can tell me what you can spend, I will see if it can be done. Second class to Pau £3 15s. 11d. There is a cheaper way by steamer from London to Bordeaux, and then by rail, second class, £2 9s. 6d. I think I could put you in the way of living there cheaply, but the address is not for publication. I should say from October to the end of May. So much harm is done by too early return to our cold spring winds.

## Figures and Tables

**Fig. 25. f1:**